# Editorial: Molecular and cellular control of B cell responses: germinal center and extrafollicular responses for cellular outputs

**DOI:** 10.3389/fimmu.2024.1354411

**Published:** 2024-01-18

**Authors:** Rinako Nakagawa, Dessi Malinova, Manuel D. Díaz-Muñoz

**Affiliations:** ^1^ Immunity and Cancer Laboratory, Francis Crick Institute, London, United Kingdom; ^2^ School of Medicine, Dentistry and Biomedical Sciences, Queen's University Belfast, Belfast, United Kingdom; ^3^ Toulouse Institute for Infectious and Inflammatory Diseases (INFINITy), Inserm UMR1291, CNRS UMR5051, University Paul Sabatier, CHU Purpan, Toulouse, France

**Keywords:** germinal center (GC) B cell, follicular T helper cells (Tfh), follicular regulatory T cell(Tfr), lymphomagenesis, memory B cell (MBC)

In the Research Topic “*Molecular and Cellular Control of B cell responses: GC and EF for cellular outputs*”, five topic-specific studies were conducted by experts in the field, focusing on mainly germinal center (GC) responses. During T-dependent (T-D) immune responses, B cells located in lymphoid follicles are activated through signals generated by the engagement of the B cell receptor (BCR) with an antigen and by interaction with cognate T cells. Activated B cells participate in either GC or extrafollicular (EF) responses. GCs form within B cell follicles in response to infection or vaccination. The original article by Riese et al., underscores the importance of the microenvironment in which B cells reside and the role of chemotaxis in B cells upon activation for establishing productive GC responses. They investigated the functions of the G protein-coupled sphongosine-1-phospate receptor type 4 (S1PR4) in B cells. S1PR4 plays a substantial role in maintaining splenic follicles and affects the responsiveness of B cells to CXCL13, a critical chemokine for B cell homing and organization within follicles. GC responses during systemic bacterial infection were significantly reduced in the absence of S1PR4 expression compared to controls, highlighting the considerable role of S1PR4 in B cell functions.

The GC provides a critical environment to facilitate two key processes: antibody (Ab) diversification and the differentiation of GC-B cells into plasma cells (PCs) or memory B cells (MBCs). The first process, known as “affinity maturation”, is essential for improving Ab affinity for an antigen. By integrating affinity maturation with the GC-B cell differentiation into PCs, the effective production of high-affinity and/or neutralizing Abs is achieved (1, 2). These Abs provide long-term protection against harmful pathogens. MBCs swiftly differentiate into PCs upon re-exposure to the pathogens, efficiently generating these beneficial Abs. In the original article, Horiuchi et al., provide insights into transcriptional regulation in MBC maintenance during T-D immune responses. By utilizing *in vitro*-induced GC-B cell system and conditional knockout (cKO) mice lacking SpiB expression in an activated B cell specific manner, they discovered that SpiB is critical for the maintenance of MBCs, which are highly likely to originate from GCs. Consequently, the number of MBCs decreases over time in cKO mice compared to controls. The absence of SpiB promotes PC differentiation by reducing the suppression of Blimp1, the essential transcription factor in PC differentiation. Nonetheless, SpiB cKO mice exhibited compromised recall responses and significantly reduced high-affinity antibody titers, underscoring the substantial roles of SpiB in long-term immunity.

In order to generate robust GC responses, the coordination among multiple cell types, including GC-B cells, follicular helper T cells (Tfhs) and follicular regulatory T cells (Tfrs), is required. Tfhs are vital for initiating and sustaining GC responses by providing essential help to B cell, while Tfrs play a critical role in preventing the expansion of autoreactive GC-B cells, which may arise as a result of somatic hypermutation (SHM) during GC responses. In their original article, Schips et al., investigated the mechanisms through which Tfrs enhance the reduction of autoreactive GC-B cells by applying the well-established computer simulation methods to consider the aspects of Tfrs. Based on the simulation results, they deduced that the number of Tfrs is limited because these cells disrupt the selection process of non-autoreactive GC-B cells through interaction with Tfhs. This study also illustrated the potential of Tfrs to effectively prevent the accumulation of autoreactive pre-PCs derived from GC-B cells.

The elaborate molecular networks within each key cell population in the GC are necessary for initiating and sustaining the functions of GC reactions. Betzler et al., have compiled a comprehensive review that examines the transcriptional network regulating the differentiation and functions of GC-B cells, Tfhs and Tfrs during GC responses, with particular emphasis on the transcriptional activator BOB.1 (a.k.a. OBF.1). The review highlights that the activated B cell-specific deletion of BOB.1. leads to the absence of GCs, followed by a detailed examination of how this transcription factor (TF) interacts with other TFs to intricately orchestrate the control of GC responses. The high rates of proliferation and programmed DNA breaks induced by SHM and class switch recombination in GC-B cells, pose a significant risk of transformation in these cells. Considering this, the authors also discuss the relationship between lymphomagenesis and the dysregulation of pivotal TFs governing GC responses, such as MYC and Forkhead Box O1 (FOXO1). Given the substantial importance of FOXO1 in B cells, Lees et al., provide an in-depth review on the current research regarding the roles of individual FOXO TF family members in B cell physiology and pathophysiology. As FOXO proteins are bona fide tumor suppressors, lymphomas thrive by inactivating their functions through mutations in many B cell malignancies. This includes mechanisms, such as enforcing nuclear localization of FOXO1, by which malignant cells enhance their survival and proliferation, thereby contributing to the promotion of lymphomagenesis. Nonetheless, FOXO1 nuclear localization triggers the death of chronic lymphocytic leukemia cells, indicating the necessity for more cancer-specific investigations to gain a deeper understanding of their regulatory mechanisms.

This Research Topic aimed to discuss current knowledge and future perspectives on molecular and cellular events influencing the functional output of GC and EF responses. The papers compiled in this topic shed light on specific aspects of pre-GC and GC reactions. Yet, a more comprehensive understanding of GC reactions is required to grasp the entire picture. Finally, we thank all the authors and reviewers for their invaluable contributions; without their efforts, the publication of this Research Topic would not have been possible.

## Author contributions

RN: Writing – original draft, Writing – review & editing. DM: Writing – review & editing. MD-M: Writing – review & editing.
